# Hsp72 Overexpression Accelerates the Recovery from Caerulein-Induced Pancreatitis

**DOI:** 10.1371/journal.pone.0039972

**Published:** 2012-07-05

**Authors:** Mariia Lunova, Eugen Zizer, Ozlem Kucukoglu, Carolin Schwarz, Wolfgang H. Dillmann, Martin Wagner, Pavel Strnad

**Affiliations:** 1 Department of Internal Medicine I, University Medical Center Ulm, Ulm, Germany; 2 Department of Medicine, Division of Endocrinology and Metabolism, University of California San Diego, San Diego, California, United States of America; University of Munich, Germany

## Abstract

**Background and Aims:**

Heat shock protein (Hsp) 72 is a molecular chaperone which is upregulated in response to a variety of stress situations and has a general cytoprotective function. Increased Hsp72 levels were implicated in protection from acute pancreatitis; a hypothesis which was not tested in a transgenic mouse model yet.

**Methods:**

To analyze the role of Hsp72 during acute pancreatitis, well-characterized transgenic animals overexpressing rat Hsp72 (Hsp72 mice) under the control of the ß-actin promoter were subjected to caerulein- and L-arginine-induced acute pancreatitis. The severity of experimental pancreatitis was determined via serum lipase levels, morphometric evaluation and quantification of pancreatic edema/inflammation.

**Results:**

Hsp72 mice displayed ∼100-times Hsp72 overexpression, but no changes in the remaining chaperones. Robust Hsp72 signal was observed in pancreatic acini, but not in islets or ductal cells. In both models, elevated Hsp72 did not protect from development of acute pancreatitis and the pancreatitis-associated lung injury, but accelerated recovery from caerulein-induced tissue injury (lower lipase levels, edema, inflammation and necrosis 36 h after caerulein administration). The observed protective function of Hsp72 in caerulein-induced pancreatitis is likely due to an attenuated NF-κB signalling.

**Conclusions:**

Hsp72 overexpression accelerates the recovery from acute pancreatitis and may represent a potential treatment strategy.

## Introduction

Acute pancreatitis (AP) is an inflammatory disorder characterized by abdominal pain and elevated levels of pancreatic enzymes in the blood with an annual incidence ranging from 4.9 to 35 per 100,000 [1]. While most of the patients suffer a mild form of this disease, 15–25% develops a severe, necrotizing course associated with a high rate of complications and a mortality of 15–20 percent [2]. Despite the clinical importance of AP, its pathogenesis remains incompletely understood and given that no specific therapy is available, its treatment focuses on correction of associated conditions and a supportive care [2].

To improve our understanding of AP, a number of animal models have been developed [3]. Among them, caerulein-induced AP represents the most widely used one [3] and takes advantage of a supramaximal induction of digestive enzyme secretion, which leads to premature enzyme activation and subsequent autodigestion of the pancreas [4]. Animal models were instrumental not only in elucidating the pathogenesis of AP, but also in identifying a number of potential treatment strategies [4]. Among them, the importance of heat shock proteins (Hsps) gained particular attention [5].

Hsps represent a family of highly evolutionary conserved proteins, which can be further subdivided according to their molecular size (from 10 kDa till 100 kDa, e.g. Hsp110, Hsp90, Hsp70, Hsp40) and contain both constitutively expressed members as well as polypeptides, whose expression increases in response to stress [6]. Although they were identified as factors counteracting the damaging effects of heat stress via maintaining a proper protein folding, they are currently known to represent versatile molecular chaperones assisting with a variety of processes such as transport and degradation of proteins, whose stress-protective functions extend far beyond the heat response [7], [8]. Hsp expression can be induced by a variety of non-toxic agents, which makes them promising therapeutic targets [9]. Hsp72 constitutes the best known stress-inducible cytoplasmic chaperone, while other Hsp family members are found in different cell compartments (such as GRP78 in endoplasmic reticulum) or are constitutively expressed (such as Hsc71) [6]. In agreement with that, Hsp72 overexpression was shown to confer protection in multiple different stress situations [10].

**Table 1:** PCR Primers (5′–3′) for Genotyping and Real-Time qRT-PCR.

Genotyping PCR Primers		
rat-Hsp72	Forward	ATTACGGGGTCATTAGTTCATAGCC
	Reverse	GTAGGAAAGTCCCATAAGGTCATGT
**Real-time qRT-PCR Primers**		
rat+ms-Hsp72-	Forward	AACGTGCTCATCTTCGACCT
	Reverse	TGGCTGATGTCCTTCTTGTG

The fact that Hsp72 is strongly elevated in most experimental AP models led to the assumption that it may play a protective role [11]. In support of that, a preconditioning with thermal stress, which results in upregulation of multiple Hsps including Hsp72 ameliorated experimental AP and this effect was offset by the administration of antisense Hsp72 oligonucleotide [12]. While these data were supported by multiple subsequent studies, others failed to confirm the protective role of Hsp72 induction in experimental AP [13]. Furthermore, the effect of isolated Hsp72 overexpression was never tested. To do that, we took advantage of an established transgenic mouse line which abundantly overexpresses Hsp72 under the control of actin promoter [14]. Interestingly, an isolated overexpression of Hsp72 does not ameliorate the development of caerulein-induced AP, but accelerates the recovery from AP via attenuation of NF-κB signalling.

## Methods

### Animal Experiments

**Figure 1:**
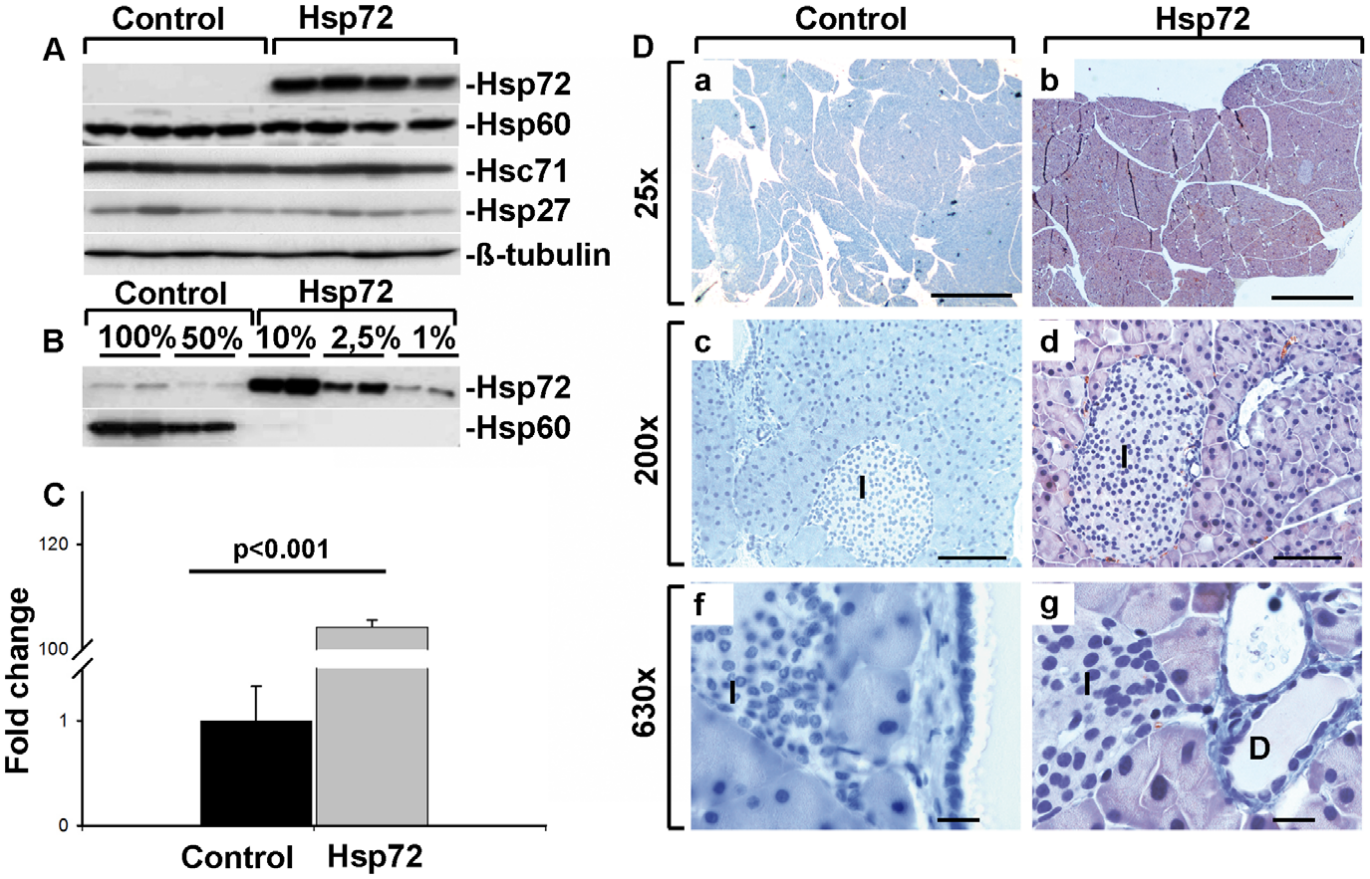
Hsp72 mice display robust Hsp72 overexpression in pancreatic acinar cells. A) Pancreatic tissue homogenates from control and Hsp72 mice were analyzed using antibodies against Hsp72, Hsc71, Hsp27, Hsp60 and ß-tubulin representing a loading control). B) Serial dilutions of pancreatic tissue homogenates were made to estimate the level of Hsp72 overexpression using two independent control and Hsp72 animals. Hsp60 was used as a loading control. C) Quantitative RT-PCR analysis of pancreatic RNA extracts confirmed the strong Hsp72 overexpression in Hsp72 mice. 4 mice per group were used and the values were normalized to the Hsp72 levels in control mice, which were arbitrarily set as 1. D) Representative tissue sections of pancreata from nontransgenic (left column) and Hsp72 mouse (right column) were stained with antibody against Hsp72. Note the robust Hsp72 overexpression in pancreatic acinar cells, but not islets (I in c,d,f,g) or ductal cells (D in g) of Hsp72 animals. Scale bars 1 mm (a,b), 100 µm (c,d) and 200 µm (f,g).

**Figure 2:**
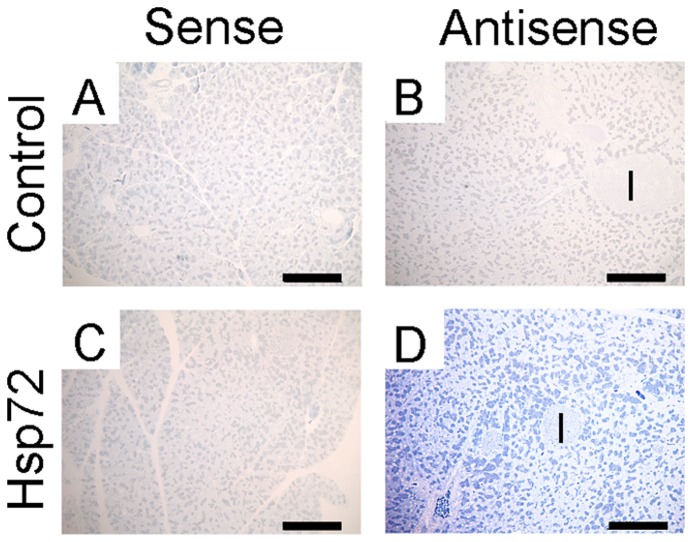
Hsp72 mice show abundant Hsp72 overexpression in exocrine pancreas. Pancreatic tissues from nontransgenic (A,B) and Hsp72 mice (C,D) were analyzed by in situ hybridization using rat Hsp72 sense (A,C) and antisense probes (B,D). Note, the prominent Hsp72 signal in exocrine, but not endocrine pancreas of transgenic animals (D), while the nontransgenic mice and sense probes did not exhibit any labelling. Scale bars 200 µm (A–D).

To study the importance of Hsp72 during experimental pancreatitis, age-matched, 4 to 5 month old mice overexpressing rat Hspa1a (Hsp72 mice) and their non-transgenic littermates (C57B6 mice) were used [14]. Genotyping of mice was performed as described [14](for primers see table 1) and pancreatitis was induced by two independent methods: caerulein and L-arginine. In the former model, six hourly injections of caerulein were administered intraperitoneally (Sigma, 100 µg/kg per mouse). L-arginine pancreatitis was induced by two injections of L-arginine-hydrochloride applied hourly (Sigma, dissolved in saline, each at a dose of 5 g/kg mouse weight) and mice were sacrificed three days later [15]. Non-treated mice were used as controls. Mice were sacrificed at indicated time-points by CO_2_-inhalation (the 6 h mice were sacrificed one hour after the sixth injection), blood was obtained by intracardiac puncture for measurement of serum lipase levels and pancreata/lung tissues were rapidly removed and cut into pieces that were: 1) immediately fixed in 10% formalin for histological / immunohistochemical analysis, 2) snap-frozen in liquid N_2_ for protein analysis, or 3) submerged into RNAlater stabilization reagent (Qiagen, Hilden, Germany) for mRNA analysis. Formaldehyde-fixed, paraffin-embedded tissues were cut into 3 µm thin sections and stained with haematoxylin and eosin (H&E). Pancreatic morphology was scored for edema, necrosis and leuko-infiltration as described previously [16]. The values are shown as means ± SEM. The animal experiments were approved by the Institutional Animal Care Committee state of Baden-Württemberg, Germany (Regierungspräsidium Tübingen) and were conducted in compliance with the German Law for Welfare of Laboratory Animals.

**Figure 3:**
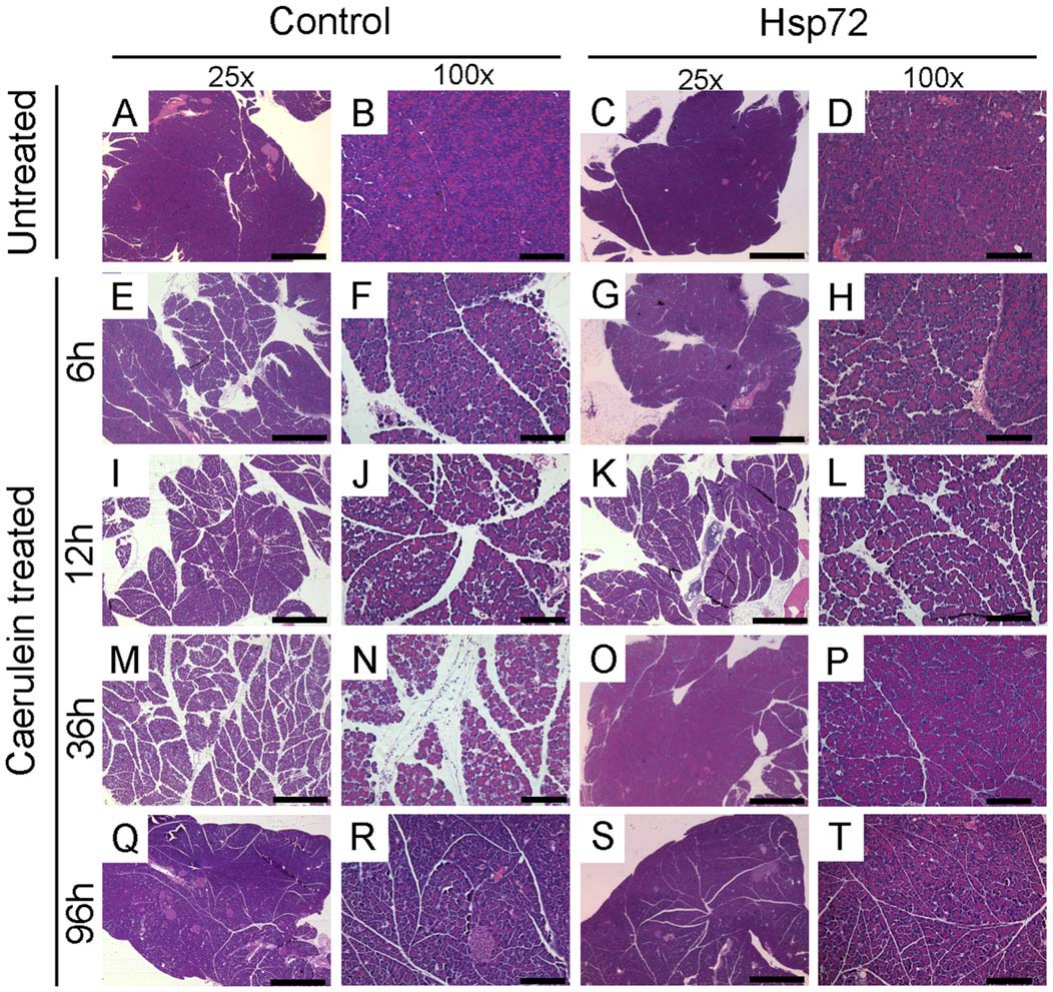
Hsp72 mice display an accelerated recovery from acute pancreatitis. Representative pancreatic tissue sections from non-transgenic (A,B,E,F,I,J,M,N,Q,R) and Hsp72 mice (C,D,G,H,K,L,O,P,S,T) were stained with hematoxylin and eosin. Animals were analyzed 6 h (E–H), 12 h (I–L), 36 h (M–P) and 96 h (Q–T) after first caerulein injection and were compared with untreated controls (A–D). Note that 36 hours after first caerulein injection, Hsp72, but not non-transgenic animals displayed an almost complete histological recovery (M–P). Scale bars 1 mm (A,C,E,G,I,K,M,O,Q,S) and 200 µm (B,D,F,H,J,L,N,P,R,T).

**Figure 4:**
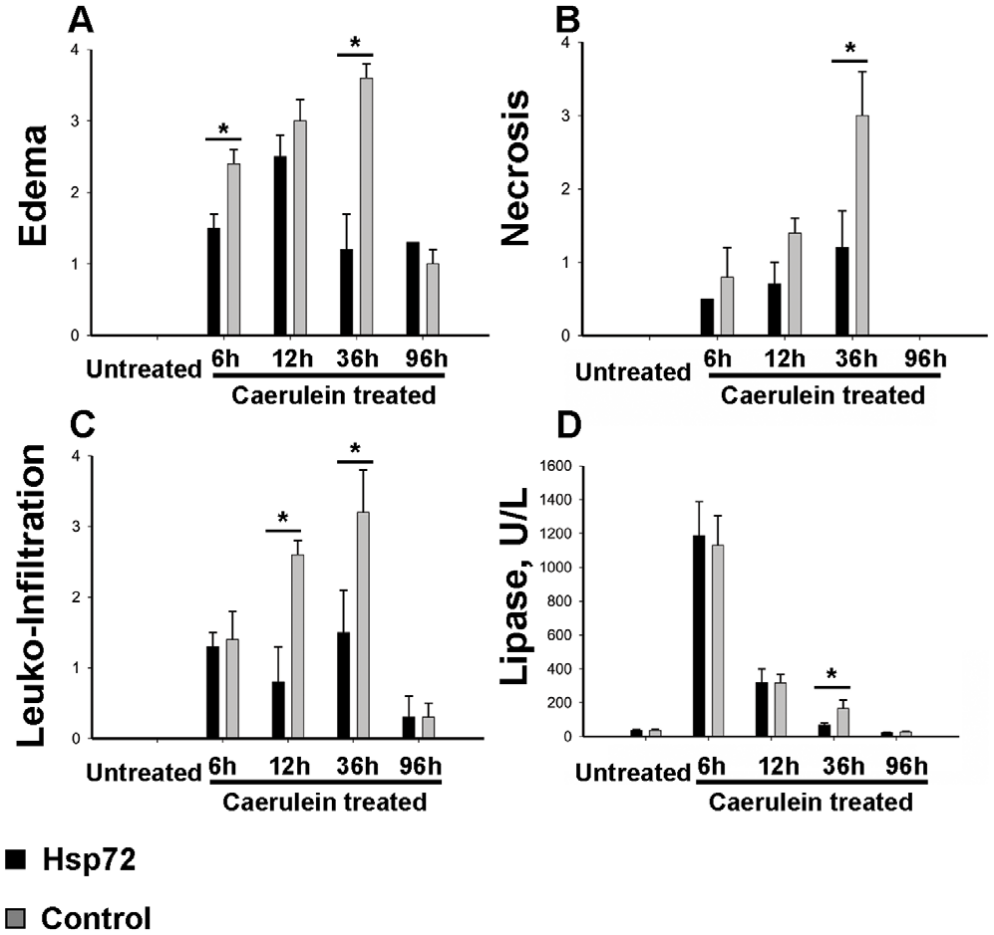
Histological scoring confirms the accelerated recovery of Hsp72 mice from caerulein - induced pancreatitis. (A–C) Animals subjected to caerulein for 6, 12, 36 and 96 hours as well as untreated animals were histologically evaluated with respect to their tissue edema (A), extent of necrosis (B) as well as leucocyte infiltration (C). Note that 36 hours after caerulein-injection, Hsp72 mice (black bars) display significantly lower values in all three assessed parameters than their non-transgenic littermates (grey bars). D) Serum lipase levels were measured in Hsp72 (black bars) and nontransgenic (grey bars) mice and visualized as mean ± SEM values from at least 4 mice per group/time point. Note that 36 hours after caerulein injection, Hsp72 mice display significantly lower lipase levels than the nontransgenic animals. *P< 0.05.

**Figure 5:**
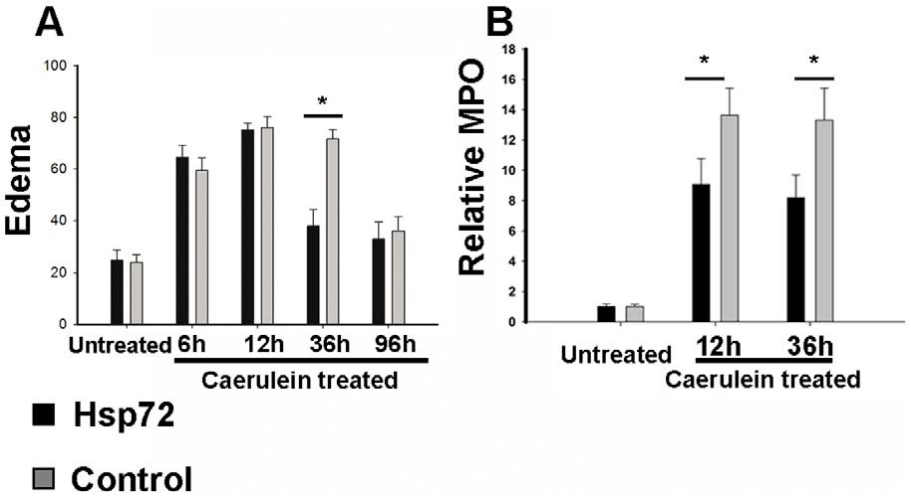
Recovery from acute pancreatitis is accelerated in Hsp72 mice. Pancreatic water content (assessed as 1-dry/wet weight) (A) and the extent of neutrophil infiltration (measured as myeloperoxidase activity) (B) was determined in untreated mice as well as animals subjected to caerulein and analyzed 6, 12, 36 and 96 hours afterwards. Results in graphs are presented as mean ± SEM from at least 4 mice per group/time point. Levels from Hsp72 and non-transgenic animals are visualized as black and grey bars, respectively. * *p*< 0.05.

**Figure 6:**
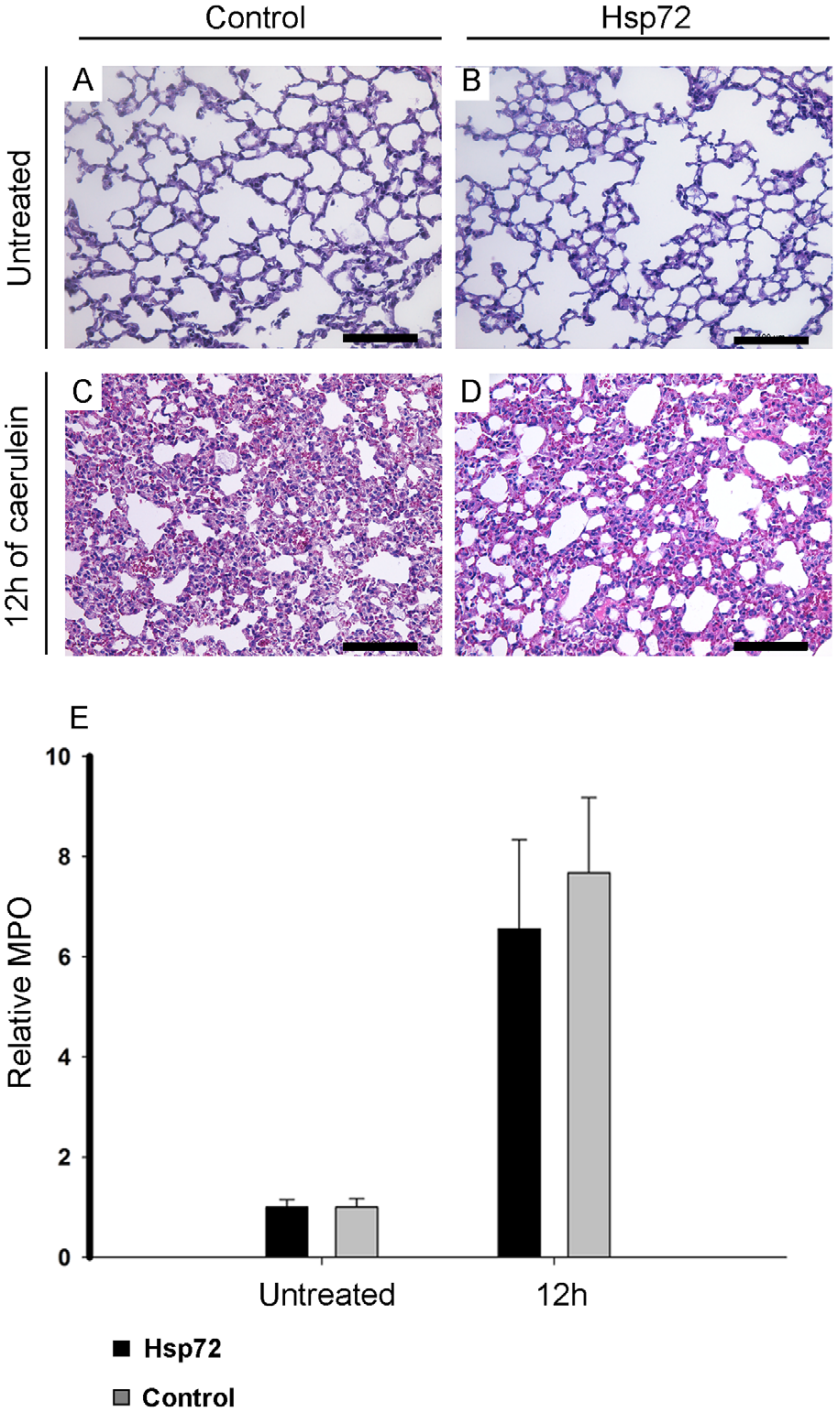
Hsp72 overexpression does not affect the development of pancreatitis-associated lung injury. Lung tissue sections from nontransgenic (A, C) and Hsp72 (B, D) mice were stained with hematoxylin and eosin. Animals were analysed 12 h after caerulein (C, D) injection and compared to untreated controls (A, B) Scale bars (A–D) 200 µm. Extent of neutrophil infiltration (measured as myeloperoxidase activity) (E) was determined in untreated mice as well as 12 hours after caerulein administration. Results are presented as mean ± SEM from at least 5 mice per group/time point. Levels from Hsp72 and non-transgenic animals are visualized as black and grey bars, respectively.

### Biochemical Methods

Total pancreas lysates were prepared by homogenization in 3% sodium dodecyl sulphate (SDS)-containing sample buffer followed by centrifugation to remove non-solubilized debris. Protein concentration was determined using the Bio-Rad DC protein assay (Bio-Rad Laboratories, Hercules, CA). Equal amounts of protein were separated by SDS-polyacrylamide gel electrophoresis (PAGE) and transferred to PVDF membranes. After incubation with the appropriate primary and secondary antibodies, the resulting HRP signal was visualized by enhanced chemiluminescence (GE Healthcare/Amersham Biosciences, Buckinghamshire, UK). Following antibodies (Abs) were used: anti Hsp72 (Ab SPA 812), anti Hsp60 (SPA 805) (both Stressgen, Ann Arbor, USA) Hsc71 (SPA 819, Stressgen, Ann Arbor, USA), Hsp27 (sc-1049, Santa Cruz Biotech, Germany), p65 (sc-372, Santa Cruz, Germany), p65 (RB1638, Neomarkers, Germany), ß-tubulin (T8328, Sigma, Germany) and HDAC2 (2540, Cell Signalling, Germany).

**Figure 7:**
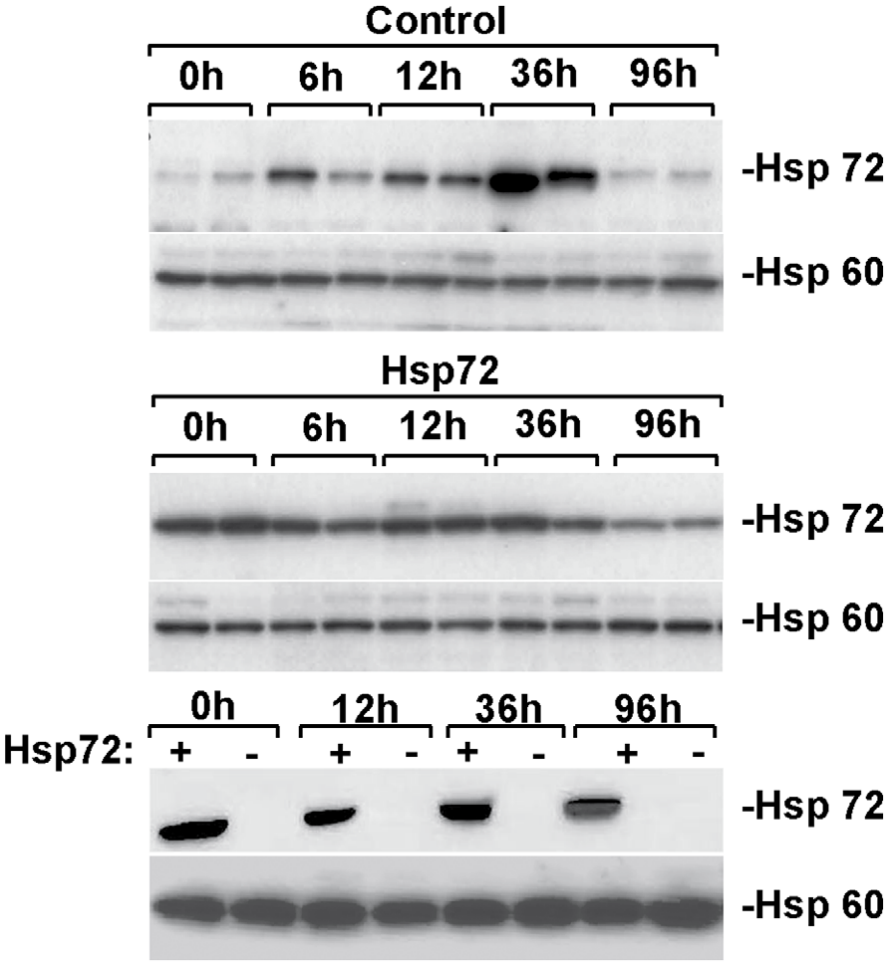
Caerulein-induced pancreatitis results in moderate Hsp72 overexpression. The level of pancreatic Hsp72 expression was assessed in animals subjected to 6 intraperitoneal injections of caerulein and sacrificed at indicated times afterwards as well as in non-treated mice. Hsp60 was used as a loading control. Note that caerulein-induced pancreatitis results in increased Hsp72 levels in non-transgenic animals, which however remain well below the levels observed in Hsp72 at all analyzed times.

#### Tissue staining

The harvested pancreata/lung tissues were incubated in 10% buffered formalin at RT overnight, embedded in paraffin, sectioned (3 µm thick), and either stained with haematoxylin and eosin for histological examination or left unstained for immunohistochemistry and in situ hybridization. The immunohistochemistry was performed with a mouse ABC Staining System (Vectastain ABC Kit, Vector Laboratories; Inc, Burlingame CA). To that end, deparaffinized sections were cooked in Antigen Retrieval Solution (Vector Laboratories, INC., Burlingame, CA) and preincubated with 3% H_2_O_2_ for 10 minutes to remove endogenous peroxidase activity. After exposure to fish skin blocking solution for 20 minutes, the slides were incubated with anti Hsp72 (Ab SPA 810, Stressgen, Ann Arbor,USA) or p65 (RB1638, Neomarkers, Germany) antibody for 1 h, washed with 0.1% Tween-20-containing Tris Base Saline buffer (TBST), and incubated with secondary antibody for 30 minutes (all at room temperature). The sections were then subjected to AB enzyme reagent for 30 minutes and washed with TBST. The peroxidase substrate Vector Nova Red was added for stain development (Vector Laboratories, INC., Burlingame, CA).

**Figure 8:**
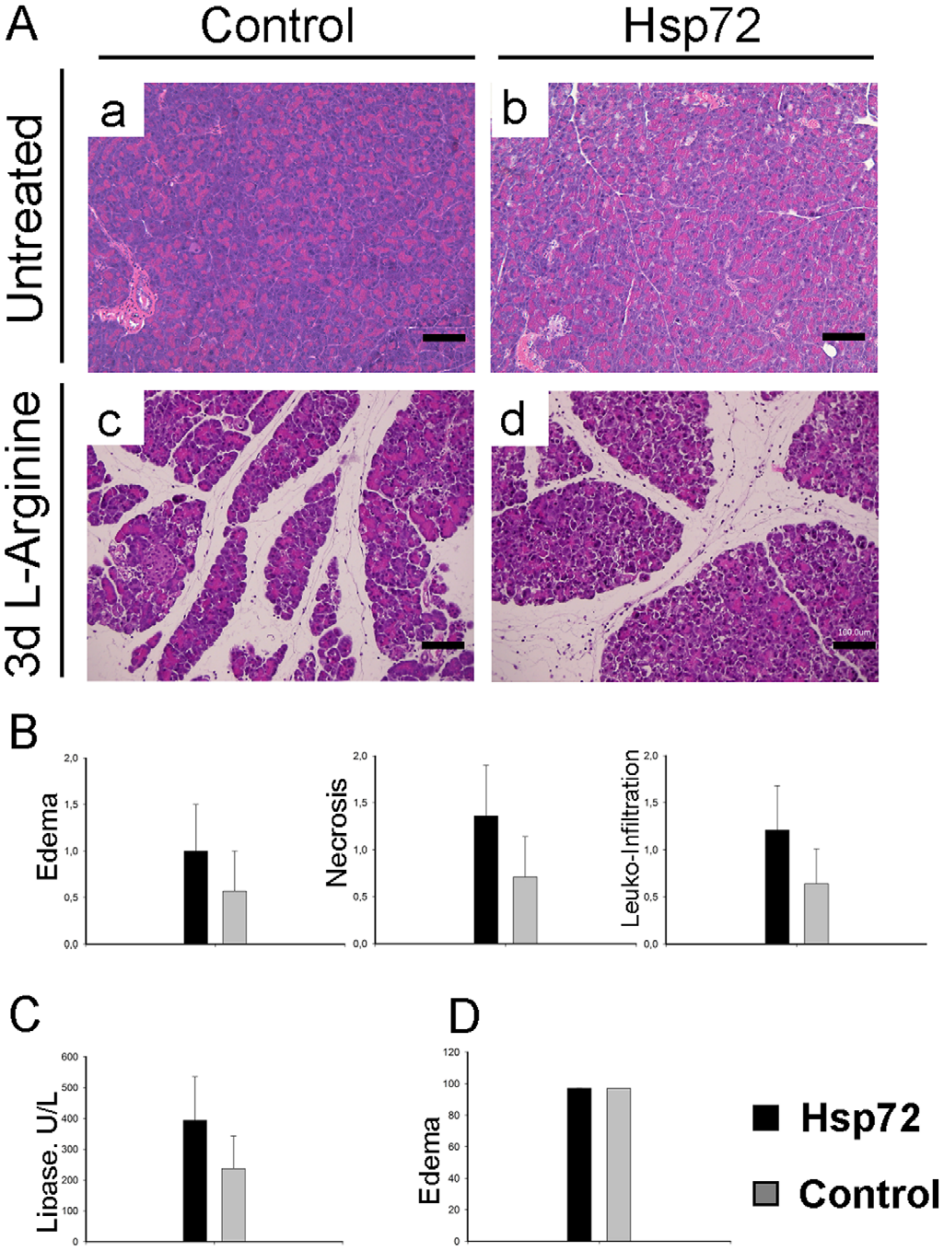
Hsp72 overexpression does not protect from L-arginine-induced acute pancreatitis. (A) Pancreatic tissue sections from nontransgenic (a, c) and Hsp72 mice (b, d) were stained with haematoxylin and eosin prior to (a,b) and after treatment with L-arginine (c, d). Scale bars (a–d), 100 µm. (B) Morphometric analysis of histological sections reveals the extent of edema, necrosis and leukocyte infiltration in nontransgenic (grey bars) and Hsp72 animals (black bars) injected with L-arginine (C) Serum lipase levels and pancreatic water content (D) determine the extent of pancreatic/acinar injury in Hsp72 (black bars) and nontransgenic mice (grey bars) subjected to L-arginine. The data are presented as mean ± SEM. At least 5 mice were used per group/time point.

Nonradioactive in situ hybridization (ISH) of paraffin-embedded sections was performed as described previously [17]. Rat Hsp 72-specific sense and antisense DNA probes were PCR amplified from rat cDNA using primers harboring the T7 recognition sequence: 5′-CTAATACGACTCACTATAGGG-GCTTCTCCCGTTTGACACTC-3′ (forward sense primer for antisense probe), 5′-CCGGACTGGTTGTTATTTGC-3′ (reverse sense primer for antisense probe), 5′-CTAATACGACTCACTATAGGG-CCGGACTGGTTGTTATTTGC-3′ (forward antisense primer for sense probe) and 5′-GCTTCTCCCGTTTGACACTC-3′ (reverse antisense primer for sense probe). DNA samples were than in vitro transcribed and digoxigenin-labeled using T7 RNA polymerase and digoxigenin RNA-labeling reagents (Roche Diagnostics, Indianapolis, USA). The antisense and sense probes (each 200 µg ml^−1^) were hybridized to deparaffinized and dehydrated tissue sections, which were previously incubated with 1% hydrogen peroxidase and digested with proteinase K. After blocking endogenous biotin (Dako Germany, Hamburg) and amplifying the signal with an anti-DIG antibody conjugated with alkaline phosphatase (Roche, Mannheim, Germany), the signal was visualized by incubating sections with NBT and BCIP (Roche).

### Edema Measurement

To determine the extent of pancreatic edema, pancreata were cleaned from the fat, weighted (i.e. wet weight) and dried at 160°C for 24 h (i.e. dry weight) [18]. The water content was determined as (wet weight-dry weight)/wet weight [18].

### MPO Activity

This was measured in pancreas and lung as described previously [19] using the commercially available MPO kit from Calbiochem, Germany.

### Nuclear Extract Preparation

Preparation of nuclear extract was performed as described previously [20]. Briefly, mouse pancreata were gently homogenised in buffer A (10 mM HEPES - KOH pH 7.9 at +4°C, 1.5 mM MgCl_2_, 10 mM KCl, 0.5 mM DTT (dithiotreithol) and 0.2 mM PMSF) and homogenates were centrifuged at +4°C, 2000 g for 10 min. Supernatant representing cytosolic fraction was collected and pellet was incubated with buffer B (20 mM HEPES - KOH pH 7.9 at +4°C, 25 % glycerol, 420 mM NaCl, 1.5 mM MgCl_2_, 0.2 mM EDTA, 0.5 mM DTT and 0.2 mM PMSF) on ice for 30 min. During the incubation samples were vortexed 3 times. Cellular debris was removed by centrifugation for 2 min 20000 g at +4°C and the supernatant representing nuclear fraction was stored at –80°C. Protein concentration was determined using the Bio-Rad DC protein assay (Bio-Rad Laboratories, Hercules, CA) and equal amounts of samples were loaded on SDS-PAGE. Proteins were transferred to PVDF membrane and blotted with the respective antibodies.

### Quantitative Real Time RT-PCR

Total RNA was isolated using RNeasy mini kit (Qiagen, Valencia, CA) and transcribed into cDNA with a Superscript II reverse transcriptase (Invitrogen, Carlsbad, CA). Quantitative real-time PCR was performed with a Sequence Detection System (Applied Biosystems 7500 fast Real Time PCR system) and specific primers (Table 1). Samples were analyzed in duplicates and at least three individual mice were tested for each genotype. L7 ribosomal protein was used as an internal control and cDNA levels were normalized so that L7 expression was approximately equal in all tested samples. After confirming that the amplification efficiency was approximately equal for all genes, the transcript levels relative to L7 were determined and reported as means ± SD.

### Statistical Analysis

The results were expressed as mean ± standard error mean (SEM). The Newman-Keuls and Fisher’s Least Significant Difference (LSD) tests were used for multi-group comparisons. Differences were considered statistically significant at *p <* 0.05.

**Figure 9:**
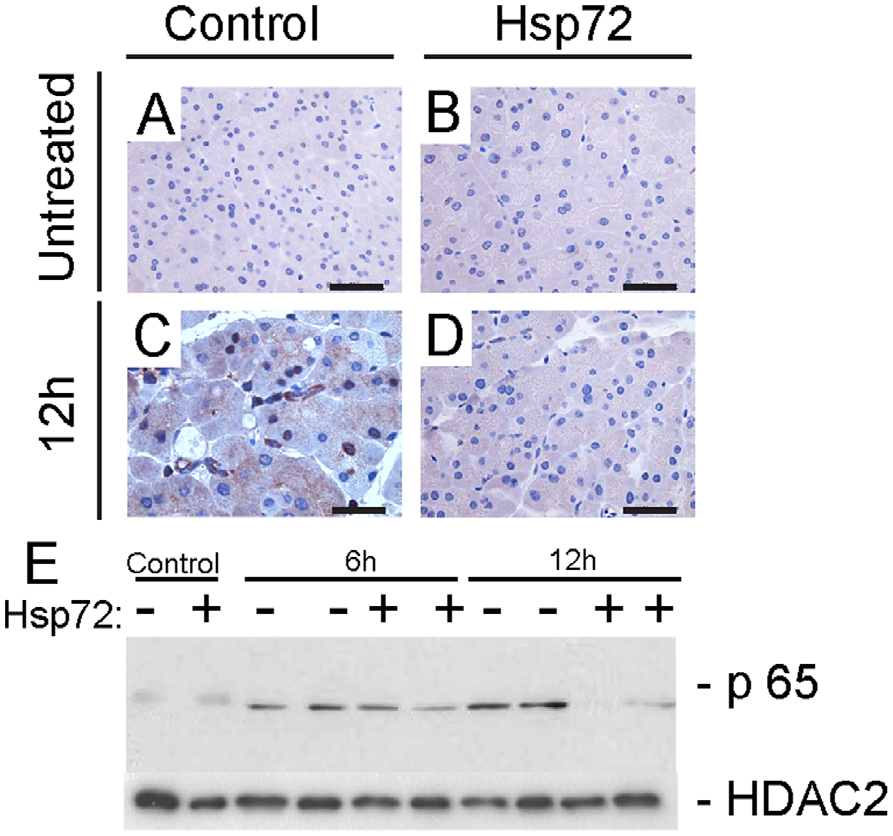
Hsp72 mice display attenuated NF-κB signalling. Immunohistochemical staining (A) depicts nuclear localization of p65 which serves as a marker of NF-κB activation. Nontransgenic (left column) and Hsp72 mice (right column) were analyzed prior to (upper row) and 12 hours after caerulein administration (lower row). Scale bar 50 µm. (B) To further quantify the extent of NF-κB activation, pancreatic nuclear extracts were incubated with p65 antibody. Note the attenuated NF-κB signalling in caerulein-treated Hsp72 mice when compared with their non-transgenic littermates. HDAC2 was used as a loading control.

## Results

### Hsp72 Mice Display a Pronounced Hsp72 Overexpression in Exocrine Pancreas

To characterize the pancreatic Hsp72 expression, Hsp72 mice and their non-transgenic littermates were subjected to real time RT-PCR and immunoblotting. Using these methods, we detected a ∼100× increase in Hsp72, both on mRNA and protein level (Figure 1A–C). Immunohistochemical staining depicted a marked Hsp72 over-expression in acinar cells, but not ductal cells or endocrine pancreata (Figure 1D–I). These data were confirmed by in situ hybridization, which displayed a prominent Hsp72 signal in exocrine, but not endocrine pancreas (Figure 2). Moreover, isolation of white blood cells with a subsequent real time RT-PCR showed a lack of Hsp72 overexpression (not shown). Of note, Hsp72 overexpression did not induce any significant changes in the other chaperone proteins such as Hsp27, 60 and Hsc71 (Figure 1A).

### Hsp72 Overexpression Accelerates Recovery from Caerulein-induced Pancreatitis

To test, whether Hsp72 overexpression is sufficient to protect from acute pancreatitis, Hsp72 mice and their non-transgenic littermates were subjected to caerulein-induced pancreatitis. Caerulein-injection led to a pronounced pancreatic edema, leucocyte infiltration and necrosis (Supplemental Figure 1), which in non-transgenic animals peaked at 12–36 hours (Figures 3–5). No lethality was observed throughout the whole experiment. Within 12 hours after injection, no dramatic differences in severity of acute pancreatitis between Hsp72 mice and there non-transgenic littermates were noted (Figures 3–5). As an additional marker of acinar cell injury, lipase levels at 6 and 12 hours after caerulein administration were virtually identical in both groups (Figure 5). Moreover, the Hsp72 overexpression did not affect the development of pancreatitis-associated lung injury as determined by histological analysis and measurement of myeloperoxidase levels, which represents an enzyme found in the infiltrating neutrophils (Figure 6). 12 hours after caerulein administration, there was only a trend towards lower leucocyte infiltration in Hsp72 vs. non-transgenic mice and this result was confirmed by the subsequent measurement of myeloperoxidase levels (Figures 4C, 5B).

36 hours after caerulein administration, Hsp72 mice displayed a significantly lower pancreatic/acinar cell injury compared to their non-transgenic littermates as evidenced by lower lipase levels, lower leukocyte infiltration, pancreatic edema and necrosis scores (Figures 3, 4). The lower extent of edema and leukocyte infiltration was confirmed by independent assays (Figure 4). 96 hours after caerulein administration, both Hsp72 mice and their non-transgenic littermates displayed a nearly complete recovery from caerulein-induced pancreatitis and no differences were seen among groups (Figures 3–5). Therefore, Hsp72 overexpression did not influence the development of caerulein-induced pancreatitis, but accelerated the recovery from this type of injury.

### Hsp72 Upregulation During Pancreatitis

To test, whether an up-regulation of endogenous Hsp72 during pancreatitis might be responsible for the surprisingly mild phenotype observed in our mice, we analyzed Hsp72 protein levels throughout the course of the experiment. As reported previously [11] Hsp72 expression in non-transgenic animals was significantly up-regulated after caerulein-administration peaking at 36 hours, while no obvious increase was seen in Hsp72 mice. However, at all analyzed time points, Hsp72 mice maintained significantly higher Hsp72 levels than their non-transgenic littermates (Figure 7). Therefore, we conclude that even a robust Hsp72 overexpression in pancreatic acinar cells is not sufficient to ameliorate the development of acute pancreatitis.

### Hsp72 Overexpression does not Ameliorate the Development of L-arginine-induced Pancreatitis

To test, whether the obtained data are disease model specific, we subjected Hsp72 mice to a second model of acute pancreatitis. After L-arginine administration, the mice developed a mild pancreatitis (Figure 8A). However, the extent of tissue injury did not differ between Hsp72 mice and non-transgenic animals as shown by morphometric evaluation and serum lipase levels (Figure 8B–C). Finally, the water content of injured pancreata was similar in transgenic and non-transgenic animals (Figure 8C).

### Hsp72 Overexpression Attenuates NF-κB Signalling

Given that activation of NF-κB signalling plays an essential role in development of experimental pancreatitis [21] and that heat shock response was suggested to modulate this pathway [13], [22], we studied the course of NF-κB activation after caerulein administration. In non-transgenic animals, activation of NF-κB signalling was apparent 6 hours after caerulein injection and peaked 6 hours later (Figure 9). In contrast, Hsp72 animals displayed milder NF-κB activation at 6 hours which quickly returned to basal levels 6 hours later. Therefore, attenuated NF-κB signalling likely contributes to the accelerated recovery of Hsp72 mice from caerulein-induced AP.

## Discussion

In our study, an overexpression of Hsp72 failed to ameliorate the development of acute pancreatitis in two different disease models, which is in contrast to previous studies suggesting a protective role of Hsp72 in AP [13]. In addition, we were unable to confirm the previously reported inhibitory effect of Hsp72 on pancreatic trypsin activation ([23]and not shown). These results are not likely due to the transgenic mouse model used, given that the employed mice display a robust overexpression of Hsp72 in the pancreas and that the same mice were shown before to be protected against multiple different stress conditions (this study, [14]). While the transgenic mice used in this study represent the most established murine Hsp72 overexpression system, it is important to note that a constitutive overexpression under a non-natural promoter used in this model clearly differs from a situation seen *in vivo*.

As mentioned previously, our data do not support the previous reports strongly suggesting a protective role of Hsp72 from development of acute pancreatitis [13]. What might be the reason for this discrepancy? While a previous report suggested that Hsp72 is not able to protect from the trypsin-induced pancreatitis, which is more severe than caerulein-induced injury [13], the extent of caerulein-induced pancreatitis in our hands seem to be comparable to the one reported by Bhagat and L-arginine induces a less severe pancreatic damage [12].

One potential explanation of these diverging results lies in the fact that all experiments up to now used a preconditioning and/or a chemical pre-treatment to increase Hsp72 levels [13]; [24] while we used a genetic model. Of note, a preconditioning/pre-treatment, particularly with heat shock, leads to a variety of changes, which are not limited to Hsp72 up-regulation [25]. In that respect, Hsp72 may be necessary, but not sufficient to protect from acute pancreatitis. For example, Hsp72 needs a variety of co-factors such as Hsp40 or nucleotide-exchange factors [26] and an isolated Hsp72 increase may therefore not be sufficient for optimal Hsp72 function. Alternatively, the Hsp72 induction seen during acute pancreatitis might be sufficient for an effective tissue protection and an additional increase in Hsp72 levels would therefore not provide any further benefit. Hsp72 knockouts will be needed to test this intriguing possibility.

Another reason for the observed data might be the fact, that our mice displayed an isolated Hsp72 overexpression in pancreatic acinar cells, but not ductal cells, endocrine pancreas and/or immune cells, whereas the previously used models likely resulted in ubiquitous Hsp72 overexpresion. While Hsp72 overexpression in our mice is driven by the actin promoter, which should enable a widespread Hsp72 overexpression, it has been noted previously that this is not always the case. For example, the mice used in our experiments were shown not to have any appreciable Hsp72 overexpression in the liver and adipose tissue [27]. Therefore, an isolated Hsp72 overexpression in pancreatic acinar cells may not be sufficient to prevent from the development of acute pancreatitis. This may indeed be the case given the established crosstalk between the exocrine and the endocrine pancreas [28] as well as the emerging importance of ductal cells in development of pancreatitis [29].

Last but not least, Hsp72 mice displayed an accelerated recovery from AP which is likely at least in part due to a milder activation of NF-κB signalling. To that end, activation of NF-κB signalling is essential for development of AP and hyperthermia was shown to delay NF-κB activation seen in caerulein-induced AP [21]. This finding is not surprising given that Hsp72 represents a chaperone essential for protection from oxidative stress and given that oxidative stress represents a prominent activator of NF-κB signalling [7].

## Supporting Information

Figure S1 Representative hematoxylin and eosin stained pancreatic tissue sections from caerulein-treated animals highlight the occasional presence of necroses (arrowhead).
(TIF)
