# Supplementary figures and images for: Hsp72 Overexpression Accelerates the Recovery from Caerulein-Induced Pancreatitis
Source: PLoS One. 2012 Jul 5;7(7):e39972. doi: 10.1371/journal.pone.0039972 (PMC3390337; doi:10.1371/journal.pone.0039972)

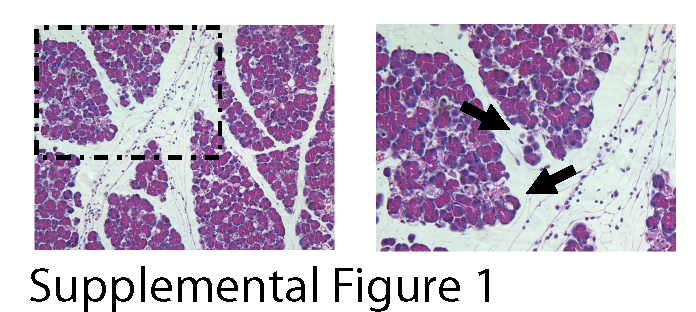

Supplement: Figure S1 — Representative hematoxylin and eosin stained pancreatic tissue sections from caerulein-treated animals highlight the occasional presence of necroses (arrowhead). (TIF) [file pone.0039972.s001.tif]
